# Intestinal barrier damage contributes to a higher prevalence of frailty in aging people living with HIV: a retrospective case control study in a Chinese cohort

**DOI:** 10.3389/fimmu.2024.1480083

**Published:** 2024-10-25

**Authors:** Xiaolei Xu, Jing Ouyang, Jiangyu Yan, Yanqiu Lu, Vijay Harypursat, Hao Wu, Yaokai Chen

**Affiliations:** ^1^ Beijing Key Laboratory for HIV/AIDS Research, Clinical Research Center for Infectious Diseases, Beijing Youan Hospital, Capital Medical University, Beijing, China; ^2^ Department of Infectious Diseases, Chongqing Public Health Medical Center, Chongqing, China

**Keywords:** aging, HIV, intestinal barrier, frailty, inflammation

## Abstract

**Background:**

It has been previously demonstrated that intestinal barrier damage is one of the underlying mechanisms leading to frailty in non-HIV-infected aging populations. However, there is a paucity of direct evidence which demonstrates the association between intestinal barrier damage and frailty in people living with HIV (PLWH).

**Methods:**

The present study is a retrospective case control study. Participants older than 50 years old were stratified into a frail/pre-frail group (case group) and non-frail group (control group) according to the Fried frailty phenotype. We collected and curated data concerning socio-demographic variables, psychological states and social functioning, and clinical information associated with the identification of biomarkers of intestinal barrier damage, microbial translocation, and levels of inflammatory cytokines of participants.

**Results:**

The case group had significantly higher levels of Reg-3α (*p*=0.042) and I-FABP (*p*=0.045) compared to the control group. We further observed, after adjusting for confounding factors by logistic regression analysis, that I-FABP levels remained significantly higher in the case group compared to the control group (*p*=0.033). Also, Fried Phenotype scores positively correlated with I-FABP levels (r_s_=0.21, *p*=0.01), LPS levels (r_s_=0.20, *p*=0.02), and sCD14 levels (r_s_=0.18, *p*=0.04). Moreover, the study confirmed both the positive correlation between inflammatory cytokines (IL-6 and IP-10) with frailty in aging PLWH, and between inflammatory cytokines (IL-6, IL-8 and IP-10) with biomarkers of intestinal barrier dysfunction in older PLWH.

**Conclusion:**

The present study indicates that the inflammation induced by intestinal barrier damage/dysfunction is likely to contribute to frailty in aging PLWH.

## Introduction

Frailty is defined as a state of increased vulnerability to stressors, at the core of which is a decline in physiological reserves or decompensation of multiple organ systems, leading to an increased risk of adverse outcomes (1–3). Frailty is related not only to advanced age, but also to chronic diseases and infections. Accumulating evidence has indicated that people living with HIV (PLWH) experience a higher prevalence of frailty compared to non-HIV-infected people (4–6). A cross-sectional study of 2028 women conducted in the United States of America indicated that the prevalence of frailty in PLWH was 17.3%, which is significantly higher than that in non-HIV-infected individuals (10.0%) (7). One prospective cohort study in the Netherlands utilized the Fried frailty phenotype score to assess the prevalence of frailty in 521 HIV-infected and 513 non-HIV-infected individuals, and observed that the prevalence of frailty in HIV-infected individuals was 10.6% and the prevalence of pre-frailty was 50.7%, which were significantly higher than prevalence rates in non-HIV-infected individuals (2.7% and 36.3%) (8). A cross-sectional study in Brazil assessed the prevalence of frailty and quality of life in 201 HIV-infected patients aged ≥50 years and observed that the prevalence of frailty in HIV-infected patients was 19.4% and the prevalence of pre-frailty was 49.3% (9); a similar study assessing patients in Canada observed that the prevalence of frailty in HIV-infected patients aged ≥50 years was 14% (10).

The underlying mechanisms which result in frailty has been investigated in the non-HIV-infected population. Previous studies have revealed that impaired intestinal permeability (11, 12) and the translocation of gut microbiota and products (13, 14) may possibly lead to an overall frail state over time. In 2017, Buford and colleagues confirmed that serum concentrations of zonulin and high-mobility group box protein (HMGB1) were 22% (*p*=0.005) and 16% (*p*=0.010) higher in older adults compared to young adults (15). Four years later, Xiong et al., further demonstrated that higher concentrations of zonulin and HMGB1 were associated with frailty in older adults (16). A systematic review which included ten case-control studies and one cohort study observed that frail older adults had significantly increased levels of serum zonulin, pro-inflammatory cytokines [tumor necrosis factor alpha (TNF-α), HMGB-1, interleukin-6 (IL-6), interleukin-1 receptor antagonist (IL1-ra), and macrophage inflammatory protein beta (MIP-1β)] than healthy controls (17). A study by Kühn indicated that oral intestinal alkaline phosphatase (IAP) supplementation may regulate the intestinal barrier and further counteract the chronic inflammatory state which leads to frailty (18). The preceding studies indicate that intestinal barrier damage is one of the key underlying mechanisms leading to frailty in non-HIV-infected older populations.

It has been previously shown that HIV infection damages and disrupts the intestinal barrier, and that intestinal barrier damage may be associated with microbial translocation (19, 20), local and systemic inflammation, and the development of non-acquired immune deficiency syndrome (AIDS)-related comorbidities (21). However, there is a paucity of direct research evidence which demonstrates the putative association between intestinal barrier damage and frailty in PLWH. The present study measured representative hematological biomarkers of intestinal barrier damage, microbial translocation, and systemic inflammation in aging PLWH (aPLWH) with or without frailty, aiming to ascertain whether intestinal barrier damage may or may not contribute to a higher prevalence of frailty in aPLWH, and to potentially reveal mechanisms which underlie the development of frailty in aging.

## Materials and methods

### Study design and participants

The present study was a retrospective case control study. HIV-infected participants were recruited from the population of PLWH being treated at Chongqing Public Health Medical Center. PLWH aged 50 or older were invited to participate this study if they were willing to provide written informed consent and were agreeable to a Fried frailty phenotype assessment between 18 August 2021 and 4 March 2022.Eligible subjects were required to satisfy all the following eligibility criteria for enrolment into our study: (1) ≥50 years old, and (2) confirmed diagnosis of HIV-1 infection. We excluded patients from the study if they: (1) had active infections requiring in-hospital treatment, such as active tuberculosis, severe pneumonia, cryptococcal meningitis, or other opportunistic infections, (2) had severe chronic non-AIDS defining disease requiring inpatient care, and (3) the blood samples were unqualified. As per the Fried frailty phenotype assessment (22), five criteria of the physical examination and presence/absence of signs or symptoms were evaluated, including unintentional weight loss, low handgrip strength, exhaustion, slow gait speed, and low physical activity. Low handgrip strength was assessed by the Jamar Plus+ hand dynamometer (Performance Health Company, Chicago, US), exhaustion was assessed by means of a questionnaire, and low physical activity was defined by weekly energy expenditure of <383kcal in men, and <270kcal in women. Participants who met one or two of the above criteria were stratified into the pre-frail group, and participants meeting three or more criteria were stratified into the frail group (22). The pre-frail group and the frail group were combined to comprise the case group in this study. Participants fulfilling none of the above criteria were stratified into the control group.

### Variables and quantitative variables

We collected socio-demographic variables, assessments of psychological states and variables of social functioning, and clinical information from all participants. Socio-demographic variables included age, sex, educational status, income, living alone or not, and smoking and alcohol consumption. Psychological states and social functioning variables included difficulties in understanding and communicating, difficulties in getting around, difficulties in self-care, difficulties in getting along with people, difficulties in life activities, difficulties in participation in society, anxiety, depression, stress, social support, and cognitive impairment. Clinical information included CD4+ T-cell counts, HIV RNA viral loads, duration of HIV infection, antiretroviral therapy (ART) regimens used, and comorbidities. All the above independent variables were acquired from the medical records of participants and by utilizing questionnaires, including the Chinese version of the Montreal Cognitive Assessment (MoCA).

Blood sample collection and storage were undertaken via standard procedures, and were identical in all cases. The serum/plasma component of whole blood samples were separated from the cellular component within six hours via centrifugation, followed by immediate storage at −80°C. Biomarkers that were detected in this study included biomarkers for intestinal barrier damage [viz., regenerating islet-derived protein-3α (REG-3α), intestinal fatty acid-binding protein (I-FABP)], biomarkers for microbial translocation [viz., lipopolysaccharide (LPS), (1,3)-β-D-Glucan (BDG), soluble CD14 (sCD14)], and inflammatory cytokines [viz., IL-6, interleukin-8 (IL-8), interferon-induced protein 10 (IP-10), transforming growth factor β (TGF-β), and TNF-α].

All of the preceding ten biomarkers were measured using enzyme-linked immunosorbent assays (ELISA). ELISA kits for serum REG-3α detection[Limit of detection(LOD): 15.6pg、mL], serum sCD14 detection(LOD: 125pg/mL), plasma IL-6 detection(LOD: 31.2pg/mL), plasma IL-8 detection(LOD: 31.3pg/mL), plasma IP-10 detection(LOD: 7.8pg/mL), plasma TGF-β detection(LOD: 5.5pg/mL), and plasma TNF-α detection(LOD: 6.23pg/mL) were commercially purchased from R&D Systems (Minneapolis, USA). The serum I-FABP detection kit(LOD: 0.156ng/mL) was purchased from the Fine Test Company (Wuhan, Hubei, China), the serum LPS detection kit(LOD: 6.25pg/mL) was purchased from the CUSABIO Company (Wuhan, Hubei, China), and the serum BDG detection kit(LOD: 0.75pg/mL) was purchased from the QCHENG BIO Company (Shanghai, China).

### Simple size determination

The sample size needed to include a minimum of 69 participants per group in order to achieve at least 80% power and an overall single-sided alpha level of 0.05, with the two groups of mean value respectively set at 11.3 and 10.6, and the standard deviation set at 1.4 (23–25) in the calculated model of the Mann-Whitney-Wilcoxon Tests. Simple size was determined using Power Analysis and Sample Size (PASS), version 15.0.5 software [PASS 15 Power Analysis and Sample Size Software (2017). NCSS, LLC. Kaysville, Utah, USA, ncss.com/software/pass].

### Statistical analysis

Categorical variables are expressed as percentages, and were analyzed via the Chi-squared test. All continuous variables are reported as median values with interquartile ranges, and differences between the case group and the control group were analyzed via the Mann-Whitney U test. The differences among the three grades of frailty(non-frailty, pre-frailty and frailty groups) were analyzed via the Jonckheere-Terpstra trend test. Spearman’s correlation coefficient test was used to analyze the correlations between continuous variables. Multivariate logistic regression analysis was employed to account for confounding factors in order to evaluate the true contribution of intestinal barrier damage to frailty. Collated data were analyzed using Statistical Package for the Social Sciences (SPSS) software, version 26.0 (IBM Corp. Released 2019. IBM SPSS Statistics for Windows, Version 26.0. Armonk, NY: IBM Corp) and GraphPad Prism software, version 8.0 (GraphPad Software, Boston, MA, USA). Statistical significance was set at *p*<0.05.

### Ethics approval

Approval for this study was granted by the Ethics Committee of Chongqing Public Health Medical Center (Approval number: 2023-002-02-KY). Informed consent to participate in this study was obtained from all participants before any trial-related activities were undertaken. The research was conducted in accordance with the Helsinki Declaration of 1975, as revised in 2008.

## Results

### Participant characteristics

A total of 151 aPLWH from Chongqing Public Health Medical Center were screened in this study. Of these, 9 patients were excluded due to the active infections or severe comorbidities, and 2 patients were excluded for lack of blood samples. Altogether, 140 eligible participants were included in this study. Female patients accounted for 37.9% of the population (n=53), and the mean age was 59.8 years (range, 50-80 years). The mean Fried Phenotype score across all participants was 0.95 (SD=1.17). There were 71 (51.7%) participants were assessed to have frailty or pre-frailty based on the Fried frailty phenotype assessment, and were stratified into the case group, and 69 (48.3%) participants were assessed to not have frailty or pre-frailty, and were therefore stratified into the control group.

The baseline characteristics of the study participants by frailty status are presented in **Table 1**. The two groups were well matched by age and gender. There was no significant difference in duration of HIV infection, sexual orientation, marital status, monthly income, education, living conditions, drug abuse, smoking, alcohol consumption, abnormal BMI, and comorbidities between the two groups. However, the case group had significantly higher HIV RNA levels (*p*<0.001), and significantly lower CD4+T-cell counts (*p*<0.001), when compared to the control group. A statistically significant difference was observed in the number of participants with HIV RNA levels below the lower limit of detection between the two groups (24 vs. 43, *p*=0.001). Additionally, significantly more participants in the case group were observed to have difficulties in getting around (*p*<0.001), social participation (*p*=0.004), and self-care (*p*=0.002) than the control group. Also, the prevalence of anxiety (*p*=0.001), depression (*p*=0.003), and stress (*p*=0.035) was calculated to be significantly higher in the case group compared to the control group.

**Table 1:** Participant characteristics.

Item		Control group(n=69)	Case group			*p*-value
			Pre-Frailty (n=54)	Frailty (n=17)	Total (n=71)	
Age(years), (median,IQR)		58.0(54.5,64.25)	57.5(54.0, 65.25)	59.0(56.5, 66.5)	58.0(55.0,65.0)	0.825
Male, (%)		43(62.3)	36(66.7)	8(47.1)	44(62.0)	1.000
MSM, (%)		7(10.1)	2(3.7)	0	2(2.8)	0.199
Marital Status	Married, (%)	55(79.7)	41(75.9)	13(76.5)	54(76.1)	0.869
	Divorced, (%)	6(8.7)	6(11.1)	1(5.9)	7(9.9)	
	Widowed, (%)	8(11.6)	7(13.0)	3(17.6)	10(14.1)	
Monthly Income (CNY)	<1000, (%)	22(31.9)	15(27.8)	9(52.9)	24(33.8)	0.914
	1000-3000, (%)	24(34.8)	23(42.6)	5(29.4)	28(39.4)	
	3000-5000, (%)	18(26.1)	14(25.9)	1(5.9)	15(21.1)	
	5000-8000, (%)	2(2.9)	1(1.9)	0	1(1.4)	
	>8000, (%)	3(4.3)	1(1.9)	2(11.8)	3(4.2)	
Education (%)	No formal education, (%)	8(11.6)	8(14.8)	2(11.8)	10(14.1)	0.789
	Elementary school level, (%)	24(34.8)	20(37.0)	7(41.2)	27(/38.0)	
	Secondary school level, (%)	33(47.8)	25(46.3)	7(41.2)	32(45.1)	
	College degree or above, (%)	4(5.8)	1(1.9)	1(5.9)	2(2.8)	
Living alone, (%)		15(21.7)	13(24.1)	3(17.6)	16(22.5)	0.910
Drug abuse, (%)		0	1(1.9)	0	1(1.4)	0.322
Smoking, (%)	Never	34(49.3)	27(50.0)	12(70.6)	39(54.9)	0.282
	Ex-smoker	18(26.1)	18(33.3)	4(23.5)	22(32.0)	
	Current smoker	17(24.6)	9(16.7)	1(5.9)	10(14.1)	
Consumption of alcohol in the past year, (%)	Never	52(75.4)	31(60.8)	11(64.7)	42(61.8)	0.443
	Monthly or less	9(13.0)	8(15.7)	3(17.6)	11(16.2)	
	2 - 4 times per month	3(4.3)	4(7.8)	0	4(5.9)	
	2 - 3 times per week	1(1.4)	2(3.9)	1(5.9)	3(4.4)	
	4+ times per week	4(5.8)	6(11.8)	2(11.8)	8(11.8)	
Abnormal BMI, (%)		32(46.4)	18(33.3)	6(35.3)	24(33.8)	0.129
Difficulties in getting around, (%)		11(15.9)	19(35.2)	13(76.5)	32(45.1)	**<0.001**
Difficulties in self-care, (%)		3(4.3)	9(16.7)	7(41.2)	16(22.5)	**0.002**
Difficulties in understanding and communication, (%)		16(23.2)	17(31.5)	9(52.9)	26(36.6)	0.083
Difficulties in social participation, (%)		49(71.0)	47(87.0)	17(100.0)	64(90.1)	**0.004**
Social support, (%)		17(24.6)	18(33.3)	6(35.3)	24(33.8)	0.416
Anxiety, (%)		26(37.7)	23(59.3)	15(88.2)	47(66.2)	**0.001**
Depression, (%)		20(29.0)	26(48.1)	12(70.6)	38(53.5)	**0.003**
Stress, (%)		15(21.7)	18(33.3)	9(52.9)	27(38.0)	**0.035**
MoCA, (%)		65(94.2)	53(98.1)	17(100.0)	70(98.6)	0.162
CD4+ T-cell count (cells/μL), (median,IQR)		290.5(189.5, 446.8)	191.0(66.3, 351.0)	78.0(9.5, 197.5)	151.0(50.0, 305.0)	**<0.001**
Nadir CD4+ T-cell count (cells/μL), (median,IQR)		166.0(65.5, 300)	118.5(40.0, 225.0)	10.0(6.0, 114.0)	87.0(22.0, 185.0)	**0.002**
HIV RNA viral load (log, copies/mL), (median,IQR)		0(0, 2.04)	3.6(0, 4.9)	4.2(1.7, 5.9)	3.8(0, 5.1)	**<0.001**
Undetected HIV RNA viral load, (%)		43(62.3)	20(37.0)	4(23.5)	24(33.8)	**0.001**
Duration of HIV infection > 10 years, (%)		2(2.9)	1(1.9)	0	1(1.4)	0.543
Impaired Glucose Tolerance, (%)		20(29.0)	21(38.9)	7(41.2)	28(39.4)	0.193
Hypertension, (%)		1(1.4)	1(1.9)	1(5.9)	2(2.8)	0.576

Categorical variables are presented as percentages and are analyzed using the Chi-squared test, whereas continuous variables are expressed as medians with interquartile ranges, and have been assessed for differences between the case and the control groups using the Mann-Whitney U test. The values in bolded red font indicates *p*<0.05.

MSM, men who have sex with men; BMI, Body Mass Index; MoCA, Montreal Cognitive Assessment; IQR, interquartile range.

### Differences in levels of intestinal barrier damage biomarkers, microbial translocation biomarkers, and inflammatory cytokines between the two groups

As shown in **Table 2**, the case group was observed to have significantly higher levels of Reg-3α (*p*=0.042) and I-FABP (*p*=0.045) than the control group, indicating that participants with frailty or pre-frailty experienced more intestinal barrier damage. However, we did not observe any significant statistical difference between the two groups where levels of LPS, BDG, and sCD14 levels (biomarkers of microbial translocation) were concerned.

**Table 2:** Comparation of biomarkers of intestinal barrier damage and microbial translocation, and inflammatory cytokines in the two groups.

Biomarkers	Control group (n=69)	Case group (n=71)	*p’*-value	*aOR (95% CI)*	*p_a_*-value
REG-3α (pg/mL), (median,IQR)	119.7(68.1,204.4)	141.5(110.4,260.8)	**0.042**	/	0.833
I-FABP (ng/mL), (median,IQR)	1.7(1.3,2.1)	2.0(1.5,2.7)	**0.045**	1.471(1.043, 2.076)	**0.033**
LPS (pg/mL), (median,IQR)	13.5(5.3,24.0)	17.3(10.2,35.3)	0.086	/	/
BDG (pg/mL), (median,IQR)	20.2(16.2,49.9)	20.1(16.0,24.0)	0.484	/	/
sCD14 (ng/mL), (median,IQR)	1140.1(571.1,1790.5)	1390.3(853.2,1851.8)	0.193	/	/
IL-8 (pg/mL), (median,IQR)	0(0,1.0)	0(0,1.9)	0.472	/	/
IL-6 (pg/mL), (median,IQR)	0.02(0,3.3)	1.9(0,5.4)	**0.002**	/	0.116
IP-10 (pg/mL), (median,IQR)	118.6(72.9,248.1)	377.0(134.6,846.8)	**<0.001**	1.002(1.000, 1.003)	**0.019**

*p*’-value were calculated via Mann-Whitney U test, while aOR and *p_a_*-value were obtained using multivariate logistic regression analysis after adjusting for difficulties in getting around, difficulties in self-care, difficulties in participation in society, anxiety, depression, stress, current CD4+ T-cell count, and current HIV-RNA viral load. The values in bolded red font indicates *p*<0.05.

REG-3α, regenerating islet-derived protein-3α; I-FABP, intestinal fatty acid-binding protein; LPS, lipopolysaccharide; BDG, (1,3)- β-D-Glucan; IP-10, interferon-induced protein 10; IQR, interquartile range; CI, confidence interval; aOR, adjusted odds ratio.

The levels of the inflammatory cytokines IP-10 (*p*<0.001) and IL-6 (*p*=0.002) were significantly increased in the case group compared to the control group, suggesting that participants with frailty or prefrailty exhibited markedly higher systemic inflammation than those without these conditions. However, no significant differences in IL-8 levels were observed between the two groups. Furthermore, the values of TNF-α and TGF-β in the majority of samples were below the detection limit, resulting in median concentrations of 0 pg/mL for both groups (data not shown). Consequently, both cytokines were excluded from the analysis in accordance with strategies employed in other studies (26, 27).

When analyses were adjusted for difficulties in getting around, self-care, social participation, anxiety, depression, stress, current CD4+ T-cell counts, and HIV RNA levels, the associations remained statistically significant for I-FABP levels (*p*=0.033) and IP-10 levels (*p*=0.019). In contrast, the observed differences in other biomarker levels (Reg-3α and IL-6) diminished after adjusting for confounders.

### Correlations between Fried Phenotype Score and levels of intestinal barrier damage biomarkers or microbial translocation biomarkers

We employed the Spearman’s correlation coefficient test to investigate the relationship between Fried Phenotype scores and intestinal barrier damage biomarkers, as well as the association between Fried Phenotype scores and microbial translation biomarkers. It illustrates a weak positive correlation between the concentrations of I-FABP (r_s_=0.21, p=0.01), an intestinal barrier damage biomarker, and Fried Phenotype scores, while Reg-3α did not exhibit a significant association with frailty (**Figures A-1A-E**). Additionally, the concentrations of microbial translation biomarkers (LPS and sCD14) (r_s_=0.20, *p*=0.02 and r_s_=0.18, *p*=0.04) were found to be associated with Fried Phenotype scores; however, the correlation between BDG and the frailty was not statistically significant.

**Figure 1:**
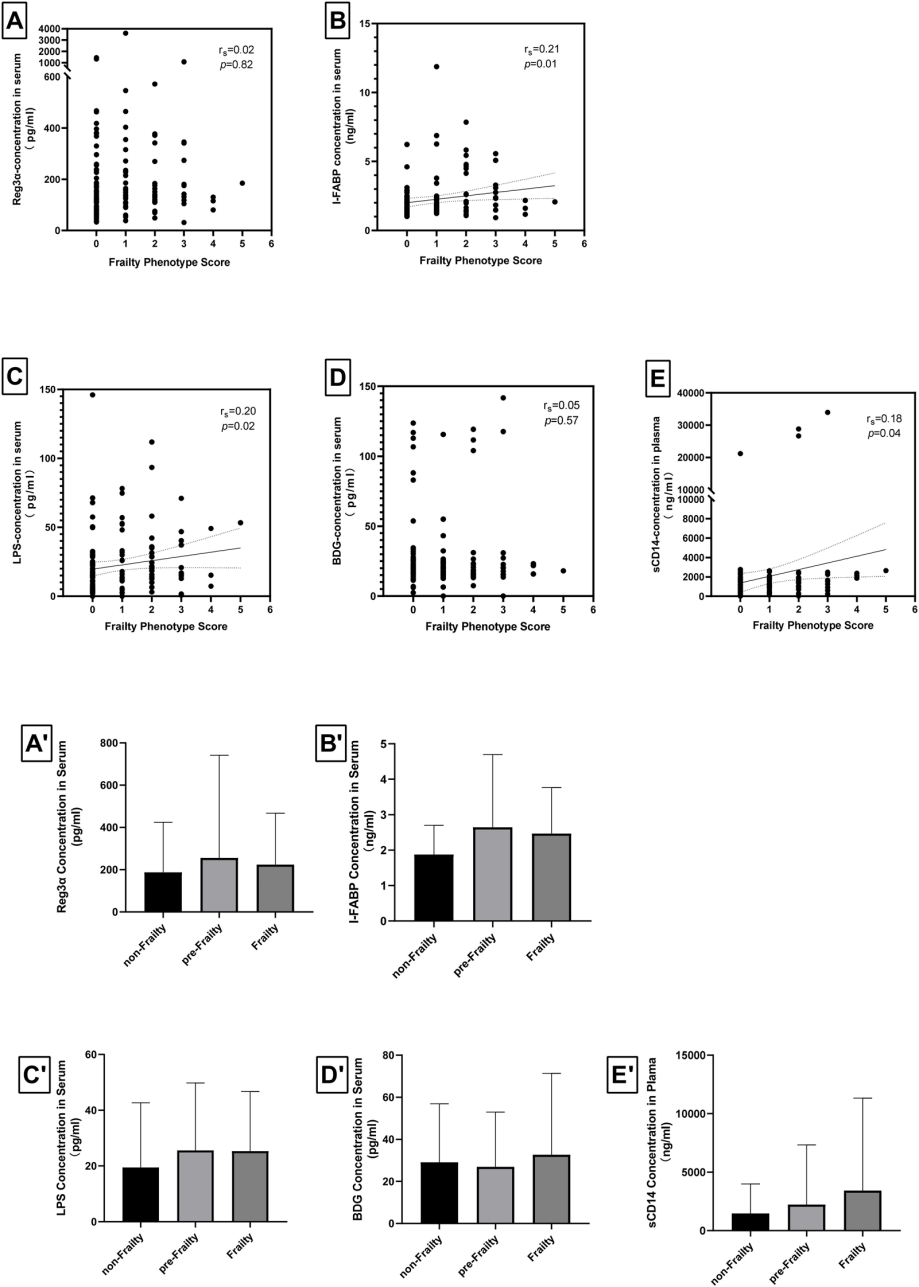
Correlations of levels of intestinal barrier damage biomarkers, levels of microbial translocation biomarkers, and Fried phenotype scores in the study cohort. The Spearman’s correlation coefficient test showed **(A)** no correlation between Fried phenotype score and REG-3α levels (r_s_=0.02, *p*=0.82), **(B)** weak correlation between Fried phenotype score and I-FABP levels (r_s_=0.21, *p*=0.01), **(C)** weak correlation between Fried phenotype score and LPS levels (r_s_=0.20, *p*=0.02), **(D)** no correlation between Fried phenotype score and BDG levels (r_s_=0.05, *p*=0.57), **(E)** very weak correlation between Fried phenotype score and sCD14 levels (r_s_=0.18, *p*=0.04). The differences among the three study groups were analyzed using the Jonckheere-Terpstra trend test, revealing **(A’)** no significant differences in Reg-3α across the groups for frailty (*p*=0.062), **(B’)** a marginally significant difference in I-FABP exist among the three groups for frailty (*p*=0.043), **(C’)** no difference in LPS across the groups for frailty (*p*=0.105), **(D’)** no differences in BDG across the groups for frailty (*p*=0.514), **(E’)** no differences in sCD14 across the groups for frailty (*p*=0.098). * *p*<0.05.

Statistical comparisons of the levels of the aforementioned biomarkers across frailty states were performed using the Jonckheere-Terpstra trend test. As illustrated in **Figures 1A’-E’**, I-FABP was the only marker that showed a statistically significant correlation to frailty states (*p*=0.043), suggesting that it may serve as a potential therapeutic candidate for frailty intervention. In contrast, other biomarker levels (Reg-3α, LPS, BDG, sCD14) did not exhibit significant differences across varying frailty states.

### Correlations between levels of inflammatory cytokines and levels of intestinal barrier damage biomarkers or levels of microbial translocation biomarkers

We subsequently explored the potential correlation between levels of biomarkers of intestinal barrier damage and levels of inflammatory cytokines, and the potential correlation between levels of biomarkers of microbial translocation and levels of inflammatory cytokines.

**Figure 2:**
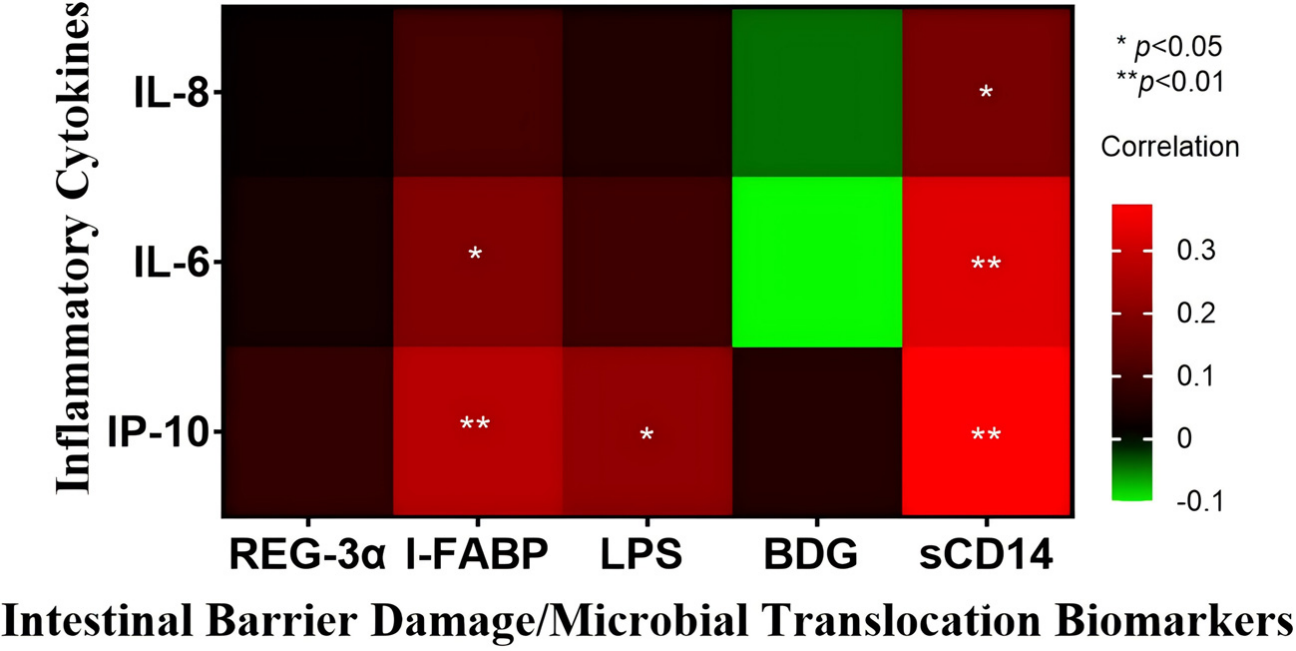
Heatmap of intestinal barrier damage/microbial translocation biomarkers and inflammatory cytokines in all included patients. All results are expressed in pg/mL, except for sCD14 and I-FABP, which are reported in ng/mL. The association between biomarkers and inflammatory cytokines was assessed using Spearman’s correlation coefficient test. * *p*<0.05, and ** *p*<0.01.

As illustrated in **Figure 2**, we observed that: (i) I-FABP levels positively correlated with IL-6 levels (r_s_=0.19 *p*=0.024) and IP-10 levels (r_s_=0.267 *p*=0.001), (ii) LPS levels positively correlated with IP-10 levels (r_s_=0.211 *p*=0.012), and (iii) sCD14 levels positively correlated with IL-6 levels (r_s_=0.324 *p*<0.001), IL-8 levels (r_s_=0.177 *p*=0.036) and IP-10 levels (r_s_=0.376 *p*<0.001).

## Discussion

In a review published in 2018, Di Sabatino et al., discussed the correlation between intestinal barrier function and frailty in the general population, and proposed the hypothesis that preservation of gut permeability and maintenance of gut microbiota composition might reduce the susceptibility to develop, or even prevent frailty-related changes (28). Several past reviews have illustrated the intricate relationship between frailty and gut anomalies. Chen et al., for example, have observed that in frail individuals, the most significant change in the gut microbiota is a decrease in the microbial (alpha) diversity of the gut microbiome (13). Picca et al., also observed that frail patients have decreased gut microbiota abundance and changes to serum concentrations of some cytokines (29). Although previous studies have reported that gut microbiota alterations in PLWH are associated with frailty, no past studies have definitively demonstrated the association between gut barrier damage/dysfunction and frailty. Our study has observed that in frail/pre-frail PLWH, the levels of gut damage biomarkers (Reg-3α and I-FABP) are higher than in non-frail participants, and even after adjusting for confounding factors, levels of I-FABP in blood remain significantly different between the two groups. Meanwhile, LPS and sCD14 levels in serum/plasma were observed to correlate with the Freid Phenotype Score. Plasma biomarkers related to intestinal barrier damage (including I-FABP and, REG3α) as well as biomarkers of microbial translocation [such as lipopolysaccharide (LPS), (1,3)-β-D-glucan (BDG), and sCD14] have been shown, in previous studies conducted by our team, to be relevant in assessing intestinal barrier function in HIV-infected individuals (30). The present study reveals the correlation between the preceding biomarkers and frailty, suggesting that a possible mechanism for the emergence of frailty in older HIV-infected individuals may be related to damage or dysfunction of the intestinal barrier.

Most often, frailty is mentioned in conjunction with inflammation, and there has been shown to be an undeniable relationship between them (31, 32). Some studies have suggested that gut biomarkers may trigger a significant immune response during HIV infection (33), causing the synthesis and release of pro-inflammatory cytokines such as IL-8 and IL-6 into the circulation (30). A systematic review by Rashidah et al., observed that frail older adults produce increased pro-inflammatory cytokines (IL-6) when compared to healthy controls (17). The present study observed both the positive correlation between inflammatory cytokines (IP-10) and frailty in aPLWH, and also between inflammatory cytokines (IL-6, IL-8 and IP-10) and biomarkers of intestinal barrier damage (as shown in **Figure 2**). In light of this, we have hypothesized that in aPLWH, the impaired intestinal barrier promotes hyper-activation of the mucosal immune system, and thus, the production of pro-inflammatory cytokines (34), which consequently fosters the emergence and development of frailty.

Although the present study found different distributions of inflammatory cytokines between case and control groups, the detected levels of IL-6 and IL-8 were lower than those in other studies. In research involving HIV-infected patients, IL-6 in plasma of PLWH prior to ART initiation (median: 2.7 pg/mL; IQR: 0.9–40.6 pg/mL) was higher than those without HIV infection (median: 1.5 pg/mL; IQR: 0.5–11.3 pg/mL), subsequently decreasing to levels lower than those of HIV-negative participants after viral suppression (median: 1.2 pg/mL; IQR:0.3–4.0 pg/mL) (35). In a study from Brazil, IL-8 levels measured in HIV-negative participants (median: 3.2 pg/mL; IQR: 2.29–4.96pg/mL) were lower than those in PLWH (median: 5.13 pg/mL; IQR: 4.26–6.48 pg/mL), although no statistically significant difference was noted between the groups (27). Given the naturally low expression of IL-6 and IL-8 in PLWH, it is important to consider potential influences from sample cryostorage and freeze-thaw cycles (36, 37), as well as anticoagulants (36–38). These factors may have contributed to values approaching zero for both IL-6 and IL-8 in this study, as illustrated in **Table 2**.

Frailty is multidimensional and complex, and some studies have classified frailty into physical frailty, psychological frailty, cognitive frailty, and social frailty according to the relevant prevailing characteristics in an afflicted patient (39, 40). In this study, the frailty assessment that we have employed primarily focuses on physical frailty, as it is frequently associated with poorer survival outcomes (41) and because the Fried frailty phenotype score utilized herein effectively evaluates the frailty status related to physical health, having been widely applied and validated in the relevant literature. Social frailty includes difficulties in understanding and communication, and difficulties in social participation and social support, and it has been reported that in the general population the incidence of social frailty is approximately 20% (42). The present study observed that in aPLWH, the incidence of social frailty [difficulties in understanding and communication (30%), and difficulties in social participation (80.7%) and social support (29.3%)] is higher than that in the general population. Also, the incidence of stress (30%), depression (41.4%) and anxiety (52.1%) is higher than that in the general older population (14%) (43). This suggests that HIV infection may exacerbate the degree of comorbid social and psychological disorders, and may well be induced by the shame, stigma, and social ostracization associated with HIV infection, and poor self-acceptance of the individual’s diagnosis after HIV infection (44–46).

The intestinal barrier is influenced by a multitude of factors, including stress and anxiety. Past research has demonstrated that chronic psychological stress and anxiety may lead to increased intestinal permeability and trigger inflammatory responses, thereby adversely impacting intestinal health. These factors may be particularly pronounced in PLWH, as mental health conditions can significantly affect the immune systems and overall well-being of these patients (47–49). In the present study, we observed a higher prevalence of anxiety, stress, and depression within the frail population; however, this difference was eliminated following multivariable statistical adjustment. Future studies must carefully consider the roles that stress and anxiety may play when investigating potential causal relationships between intestinal barrier damage and frailty.

Frailty in PLWH is also associated with prevailing HIV viral loads and CD4+ T-cell counts (which represents severity of HIV infection) (32), and our results in the present study concur with this, indicating that uncontrolled HIV replication and impaired immune function may increase the risk of development of frailty. Nadir CD4+ T-cell counts between the two groups of participants also showed a statistically significant difference, suggesting that the severity of HIV infection may also influence the development of the frailty.

We acknowledge that our study has several limitations. Firstly, the sample size is relatively small and has been derived from a single hospital, and this may impact the statistical power and reliability of the results of our study. Also, as this was a preliminary study, the inclusion and exclusion criteria were not prohibitively strict. To obtain more accurate results which focus specifically on the relationship between intestinal barrier damage and frailty, it would have been beneficial to mitigate certain confounding factors, such as the duration of HIV infection, HIV RNA viral loads, and CD4+ T-cell counts. Appropriate control of these factors may more lucidly explain the association between frailty and intestinal barrier damage. Some samples used for the present study were stored for an extended period, potentially resulting in protein degradation, which renders the low-expressing biomarkers used for this study undetectable, and leading to potential effects related to statistical correlations utilized in this study. As the present study is a retrospective case control study, we inevitably expect some recall bias, and thus prospective cohort studies in this specific field of research are required to validate our findings.

In conclusion, frailty may be considered as a comprehensive, intuitive clinical syndrome for aging persons which may potentially lead to higher hospitalization risk and substantial additional healthcare costs (50, 51). The present study indicates that the inflammation induced by intestinal barrier damage that is observed in HIV-infected individuals is likely to contribute to frailty in older PLWH. Our observations in this study may possibly contribute to the development of novel interventional options for the mitigation of manifestations of frailty, to further extend the lifespans of aPLWH, and to improve the overall quality of life of individuals with HIV infection.

## Data Availability

The original contributions presented in the study are included in the article/supplementary material. Further inquiries can be directed to the corresponding authors.
